# Prior perineural or neonatal treatment with capsaicin does not alter the development of spinal microgliosis induced by peripheral nerve injury

**DOI:** 10.1007/s00441-020-03285-8

**Published:** 2020-09-22

**Authors:** Ivett Dorina Szeredi, Gábor Jancsó, Orsolya Oszlács, Péter Sántha

**Affiliations:** Department of Physiology, University of Szeged, Dóm tér 10, Szeged, H-6720 Hungary

**Keywords:** Peripheral nerve injury, Microglia, C-fiber afferent, Myelinated afferent, Capsaicin

## Abstract

Peripheral nerve injury is associated with spinal microgliosis which plays a pivotal role in the development of neuropathic pain behavior. Several agents of primary afferent origin causing the microglial reaction have been identified, but the type(s) of primary afferents that release these mediators are still unclear. In this study, specific labeling of C-fiber spinal afferents by lectin histochemistry and selective chemodenervation by capsaicin were applied to identify the type(s) of primary afferents involved in the microglial response. Comparative quantitative morphometric evaluation of the microglial reaction in central projection territories of intact and injured peripheral nerves in the superficial (laminae I and II) and deep (laminae III and IV) spinal dorsal horn revealed a significant, about three-fold increase in microglial density after transection of the sciatic or the saphenous nerve. Prior perineural treatment of these nerves with capsaicin, resulting in a selective defunctionalization of C-fiber afferent fibers failed to affect spinal microgliosis. Similarly, peripheral nerve injury-induced increase in microglial density was unaffected in rats treated neonatally with capsaicin known to result in a near-total loss of C-fiber dorsal root fibers. Perineural treatment with capsaicin per se did not evoke a significant increase in microglial density. These observations indicate that injury-induced spinal microgliosis may be attributed to phenotypic changes in injured myelinated primary afferent neurons, whereas the contribution of C-fiber primary sensory neurons to this neuroimmune response is negligible. Spinal myelinated primary afferents may play a hitherto unrecognized role in regulation of neuroimmune and perisynaptic microenvironments of the spinal dorsal horn.

## Introduction

Damage to peripheral nerves is often inflicted by common injuries, metabolic disorders, environmental toxins, or therapeutic medications. Functional recovery may be satisfactory after nerve lesions, but the occurrence of neuropathic symptoms is not infrequent. The pathomechanism of nerve injury-induced pain in man and alterations in nociceptive behavior in experimental animals are complex. Recently, changes in the microenvironment of the neuronal circuitry in the spinal dorsal horn, in particular, an increase in microglial cell density, have gained pivotal importance. Although early studies have demonstrated a substantial increase in microglial density of the spinal cord following peripheral nerve injury (Gilmore and Skinner 1979), only more recent studies have provided clues for the understanding of the mechanisms and significance of microgliosis in the development of neuropathic pain (Sandkühler 2009; Ji et al. 2013).

Available experimental evidence indicates the significance of microglial activation and microglia-derived chemical messengers in the development of neuropathic pain, or pain-related behavior. In animal models of neuropathic pain, inhibitors of microglia activation, e.g., by minocycline, reduced mechanical hypersensitivity and activation of p38 mitogen-activated protein kinase (MAPK) in microglia (Zhuang et al. 2007). Although several chemokines and tissue mediators, including fractalkine (CX3CL1) (Old and Malcangio 2012), CCL2 (Thacker et al. 2009), CCL21 (Biber et al. 2011), and ATP (Tsuda et al. 2003; Beggs et al. 2012), have been implicated in the induction or maintenance of microgliosis, recent findings indicated a pivotal role of peripheral nerve injury-induced central release of colony stimulating factor 1 in the activation of spinal microglia (Guan et al. 2015). Despite the wealth of information on possible factors inducing microglia activation, the exact neuronal source of these agents is unclear. The expression of chemokines in injured dorsal root ganglion neurons is well documented, but the type(s) of primary afferent fibers that release these agents have not been unequivocally identified. Recent findings demonstrated that nerve injury-induced activation of myelinated but not unmyelinated sensory nerve fibers resulted in an increase in microglia density in the spinal cord within a time frame of 2 days (Suter et al. 2009). Although these findings demonstrate that activation of myelinated afferent fibers is crucial for the initiation of the microglial response after injury, they do not exclude the possibility that unmyelinated primary afferents contribute to the development of the response in some other ways. C-fiber primary sensory neurons undergo complex and prolonged processes of phenotypic and degenerative changes following injury to their peripheral axons (Fitzgerald and Woolf 1982; Petsche et al. 1983; Aldskogius et al. 1985; Such and Jancsó 1986; Jancsó 1992; Hökfelt et al. 1994; Kawasaki et al. 2008). Some of these changes, such as depletion, and possibly central release of neuropeptides (Gamse et al. 1982; Jancsó and Lawson 1987) and fluoride-resistant acid phosphatase/thiamine monophosphatase (Gerebtzoff and Maeda 1969; Jancsó and Knyihár 1975; Jessell et al. 1979; Ainsworth et al. 1981; Gibson et al. 1982; Knyihár-Csillik et al. 1989; Castro-Lopes et al. 1990; Oszlács et al. 2015), may occur simultaneously with a selective inhibition of axonal conduction in unmyelinated nerve fibers following the application of capsaicin, the archetypal transient receptor potential vanilloid 1 (TRPV1) receptor agonist onto peripheral nerves (Fitzgerald and Woolf 1982; Petsche et al. 1983; Such and Jancsó 1986).

To examine the role of specific classes of (afferent) nerve fibers in inducing spinal microgliosis, experimental tools are needed that permit an apparently total and selective elimination of a specific population of sensory nerve fibers without affecting autonomic or motor nerves. The use of the selective sensory neurotoxin capsaicin (Jancsó et al. 1977; Jancsó and Király 1981; Jancsó et al. 2012), a TRPV1 agonist (Caterina et al. 1997) proved to be a reliable experimental approach for the selective defunctionalization or even elimination of C-fiber afferent nerve fibers (Jancsó et al. 1977; Jancsó and Király 1980; Chung et al. 1985; Ritter and Dinh 1988). Systemic or local administration of capsaicin produces a rather selective loss of C-fiber primary afferent neurons and/or spinal primary afferents (Jancsó et al. 1977; Jancsó and Király 1980; Fitzgerald and Woolf 1982; Nagy and Hunt 1983; Jancsó et al. 1987; Jancsó and Lawson 1990; Pini et al. 1990; Holzer 1991; Nagy et al. 2004; Jancsó et al. 2012). Indeed, following a systemic administration of capsaicin to newborn rats, up to 95% of unmyelinated axons are lost in spinal dorsal roots (Nagy and Hunt 1983). Similarly, perineural application of capsaicin results in a complete defunctionalization of nociceptive sensory nerve fibers associated with a significant delayed loss of unmyelinated sensory axons in the treated peripheral nerve (Jancsó and Lawson 1990; Pini et al. 1990). Hence, these data clearly indicate that the selective neurotoxic effect of capsaicin on C-fiber primary sensory neurons can be exploited to study the participation of this particular class of neurons in physiological and pathological processes. In fact, in the past decades, hundreds of studies utilized this approach to study the contribution of capsaicin-sensitive C-fiber primary sensory neurons to nociceptive and other homeostatic functions (for reviews, see, e.g., Buck and Burks 1986; Jancsó et al. 1987; Maggi and Meli 1988; Szállási and Blumberg 1990; Holzer 1991; Szolcsányi 1996; Nagy et al. 2004; Jancsó and Sántha 2015). Previous studies furnished circumstantial evidence for the role of activation of myelinated but not unmyelinated sensory nerve fibers in the development of spinal microgliosis associated with peripheral nerve injury (Suter et al. 2009). Nerve lesions, in particular the most common traumatic nerve injuries, produce degenerative and lasting neuroplastic processes in the affected primary sensory neurons, which also involve central changes, including spinal microgliosis. The potential contributions of the two major disparate classes, the myelinated and the unmyelinated primary afferent fibers to the mechanisms of the spinal microglial reaction have not been investigated. This is an important issue, however, not only as regards the mechanism of spinal microgliosis, but also with respect to development of potential therapeutic approaches for treating adverse consequences of nerve injuries, such as neuropathic pain. Therefore, the present experiments were initiated to explore whether a selective functional and/or physical elimination of C-fiber primary afferents, by making use of the selective neurotoxic action of capsaicin, would significantly affect the spinal microglial reaction elicited by peripheral nerve injury. We found that selective defunctionalization and/or ablation by capsaicin of C-fiber primary afferent neurons did not significantly affect peripheral nerve injury-induced spinal microgliosis, suggesting a predominant role of myelinated primary afferents in this phenomenon.

## Materials and methods

The experiments were approved by the Ethical Committee for Animal Care at the University of Szeged in accordance with the European Communities Council Directive of 24 November 1986 (86/609/EEC) under the identifier XIV/2970/2016. All efforts were made to minimize the number of animals used and their suffering.

### Animals and surgery

#### Animals

Experiments were performed on adult male Wistar rats weighing 300–350 g. Animals were kept at a 12/12 h light/dark cycle with free access to standard chow and water ad libitum.

#### Nerve transection and ligation

The reactions of cutaneous and muscle primary afferent neurons to peripheral nerve injury are distinctly different. Whereas many cutaneous primary sensory neurons are lost, muscle primary afferent neurons are spared after peripheral nerve injury (Hu and McLachlan 2003). Therefore, two models of nerve injury involving a mixed (sciatic) and a cutaneous (saphenous) nerve containing both cutaneous and muscle and only cutaneous afferent fibers, respectively, were used in this study. The right sciatic or saphenous nerve was transected and ligated in control and capsaicin pretreated rats. Animals were anesthetized with an intraperitoneal injection of a mixture of ketamine (100 mg/kg) and xylazine (10 mg/kg). The right sciatic or the saphenous nerve was exposed high in the thigh and transected and ligated. The wound was closed, and the animals were returned to the animal house. Sham-operated animals (exposure of the respective nerve) served as controls.

#### Neonatal capsaicin treatment

This was performed as described previously (Jancsó et al. 1977). Briefly, two-day-old newborn rats were anesthetized with isoflurane and given a subcutaneous injection of capsaicin at a dose of 50 mg/kg. At the age of two months, rats were anesthetized with ketamine (100 mg/kg) and xylazine (10 mg/kg) and the right sciatic nerve was transected and ligated. Animals were sacrificed 14 days later.

#### Perineural capsaicin treatment

This was performed as described previously (Jancsó et al. 1980). Briefly, rats were anesthetized with an intraperitoneal injection of a mixture of ketamine (100 mg/kg) and xylazine (10 mg/kg). The right sciatic nerve was exposed high in the thigh, and a piece of gelfoam moistened with a 1% solution of capsaicin was placed around the nerve. Then, 30 min later, it was removed, the wound was closed and the animals were returned to the animal house. One group of rats treated perineurally with capsaicin was sacrificed 14 days later, whereas another group of rats was subjected to transection of the right sciatic nerve. These latter animals were sacrificed 14 days later. Sham-operated rats (application of the vehicle) served as controls.

### Lectin- and immunohistochemistry

Fourteen to 28 days after surgery, rats were terminally anesthetized with thiopental and perfused with 4% paraformaldehyde in 0.1 M phosphate buffer (pH 7.4). The lumbar spinal cord and lumbar spinal ganglia (L3–L5) were removed, post-fixed for 2 h, and cryoprotected overnight in phosphate-buffered saline (PBS) with 30% sucrose added. Then, 20-μm-thick transverse sections of the lumbar spinal cord were cut using a freezing microtome and processed for histochemistry. Free-floating sections were washed in PBS and incubated overnight with combinations of mouse monoclonal anti-CD11b (OX42) antibody (1:1000, Abd Serotec/Bio-Rad, Hercules, California, USA) and biotin-conjugated Bandeiraea simplicifolia isolectin B4 (IB4) lectin (1:500, Sigma-Aldrich, Saint Louis, USA) in PBS with 1% Triton X100 (TPBS). Sections were rinsed in PBS (3 × 5 min) and incubated in a mixture of Cy3-conjugated donkey anti-mouse antibody (1:500, Jackson ImmunoResearch, West Grove, PA, USA) and FITC-conjugated ExtrAvidin (1:500, Jackson ImmunoResearch, West Grove, PA, USA) in TPBS. Sections were incubated for 2 hours at room temperature, rinsed in PBS (3 × 5 min), mounted on slides, and covered with Prolong Gold mounting medium (Invitrogen, Carlsbad, California, USA).

### Image capture and quantitative analysis

The unbiased sampling of data for morphometric analysis was ensured at the following multiple stages of the experiments: (1) spinal cord sections were selected by a systematic random way from series of sections collected from the L3/L4 spinal cord segments; (2) thin optical sections (3 from each spinal cord section) used to measure microglia profile density were also selected in a systematic random way from Z-stack image series consisting of 12–15 optical sections; (3) standard sized ROI areas were also randomly selected in the optical sections following identification of the boundary between the laminae II and III of the spinal cord. In the selected ROI areas, the relative proportions of the surface areas occupied by the OX42-positive microglial profiles were determined automatically with the image analysis software after thresholding and binary segmentation (see subsequent texts for technical details).

Sets of Z-stack optical sections were captured on a Zeiss LSM 700 confocal laser scanning microscope. Image analysis was performed with ImagePro software (MediaCybernetics, Rockville, MD, USA). To estimate changes in spinal microglial cell density, the method of Blackbeard et al. (2007) was adapted and slightly modified for use on 1 μm thin optical sections captured with the confocal microscope.

In experiments involving the saphenous and the sciatic nerve, the third and the fourth lumbar segments, respectively, were used for image analysis. Average microglial profile density was determined by measurements on 3 optical sections obtained from each of the 3 sections per spinal segment analyzed per animal. High magnification z-stack serial images of the spinal dorsal horn were captured at 2 μm intervals. In order to ensure reproducible results, the parameters of the expositions were adjusted by using the real-time histogram menu of the ZEN program. After setting standard laser intensity, the sensitivity was adjusted until the peak of the background pixel intensity reached a value of 8–10. To determine the threshold pixel intensity for the identification (and later segmentation) of immunolabeled microglial profiles, pilot studies were performed. Original images of OX42 labeled spinal cord sections counterstained with the nuclear stain DAPI were converted to binary images by using the thresholding function of the ImagePro image analysis software. The threshold values for the image segmentation were set on 2 to 6 times of the peak values of the background pixel intensity distributions. As expected, the total area of the suprathreshold pixels within a standard sized counting frame was reduced continuously due to the stepwise increment of the threshold value. A similar approach was used on images showing nuclear staining for the segmentation of DAPI labeled microglial cell nuclei. The binary images representing the OX42 and the DAPI channels and segmented by the same threshold criteria were merged, and the number of double labeled pixels was determined. By increasing the threshold values, the number of double-labeled pixels reduced drastically, indicating an increasingly better separation of the pixels showing either cytoplasmic OX42 labeling, or cell nuclei stained with DAPI. This decrease was observed up to 5 times the peak of background intensity threshold. Choosing higher values further reduced the areas showing either of OX42 or DAPI positivity, and the separation was followed by the appearance of empty regions showing neither OX42 nor DAPI positivity indicating an over reduction. By applying this procedure, it was possible to prevent over- or underestimation of the OX42-immunoreactive area. Therefore, during the measurements, 5 times the peak of background intensity threshold was used for the determination of the relative cross-sectional area of OX42 positive microglial profiles. For the morphometric determination of the area covered by OX42 positive microglial profiles (microglial cell density), three standard-sized (A = 2500 μm^2^) counting frames were randomly positioned over Rexed’s laminae I–II and III–IV, respectively, in single optical sections of the dorsal horn. Microglial cell densities were measured separately in the most superficial layers of the spinal dorsal horn (Rexed’s laminae I and II), and in an area involving laminae III and IV which extends ~ 250 μm ventrally from the ventral border of lamina II as defined by the ventral border of the IB4-positive band of the substantia gelatinosa. The medio-lateral extensions of the central projection areas of treated peripheral nerves were identified by visualization of the IB4 staining. The density of OX42-positive microglial profiles was determined as percent values of the relative areas covered by OX42-labeled profiles compared to the total area of the counting frames. Data on the average microglial profile densities were obtained from 4 rats in each experimental group. The mean ± SD of the relative surface areas of microglia profiles were compared by using two-way ANOVA followed by Fisher’s post hoc comparison, with a *p* < 0.05 level of significance.

#### Drugs

Capsaicin was from Sigma-Aldrich (Saint Louis, USA). A stock solution of 1% capsaicin was prepared in a vehicle containing 6% ethanol and 8% Tween 80 in saline. For injections of newborn rats, the stock solution was diluted with saline to inject the appropriate dose in a volume of 0.1 mL. Thiopental was from B. Braun Medical S.A. (Barcelona, Spain), ketamine (Calypsol, ketamine hydrochloride) from Gedeon Richter (Budapest, Hungary), and xylazin (CP-Xylazin) from Produlab Pharma B.V. (Raamsdonksveer, The Netherlands).

## Results

### Effect of nerve transection on the distribution and density of spinal microglial cells

**Fig. 1:**
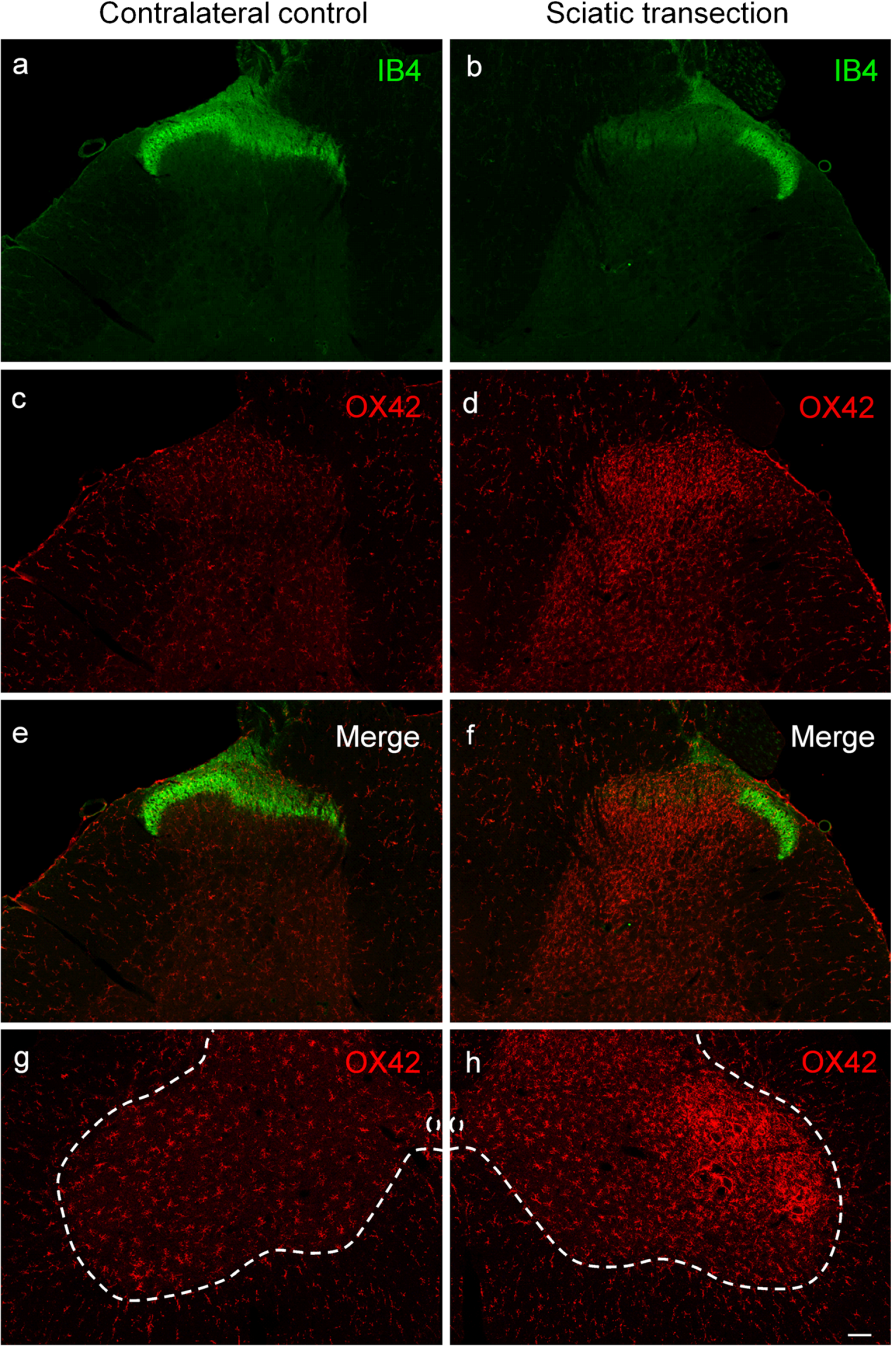
Fluorescent photomicrographs illustrating IB4 (green) and OX42 (red) staining in the spinal dorsal or ventral horn on the control side (**a**, **c**, **e**, **g**) and ipsilateral to transection (**b**, **d**, **f**, **h**) of the sciatic nerve in the L4 segment of the spinal cord. Note the disappearance of IB4 staining of C-fiber primary afferent terminals from somatotopically relating projection areas of the sciatic nerve (**b**, **f**). The increase in microglial density is confined to this area in laminae I–II and to regions of the deeper laminae III–IV just ventrally to the “IB4 gap.” Note the intense perineuronal microglia reaction around sciatic motoneurons in the ventral horn of the spinal cord ipsilateral to sciatic nerve transection (**h**). The scale bar in (**h**) indicates 100 μm and holds for all photomicrographs

**Fig. 2:**
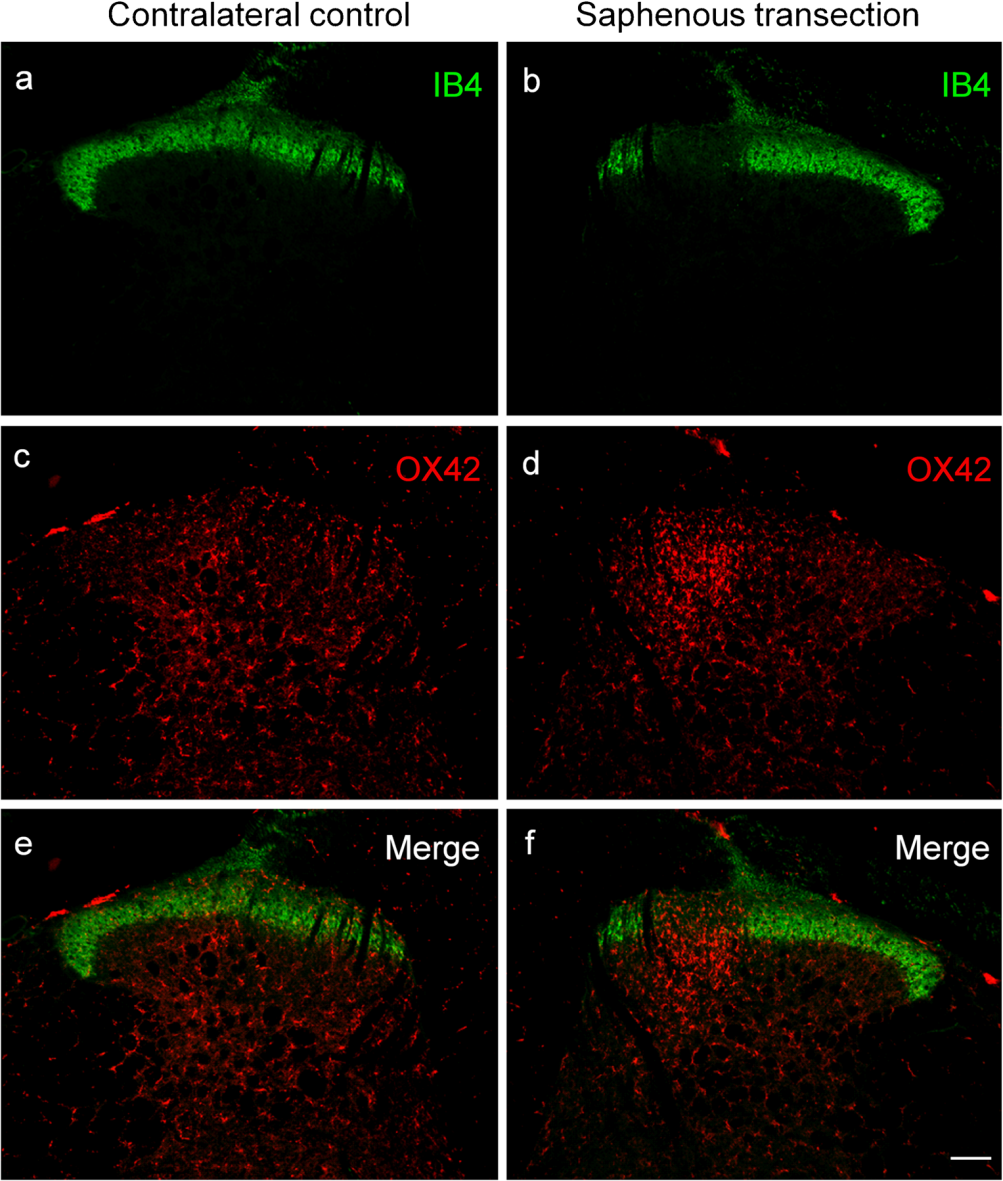
Fluorescent photomicrographs illustrating IB4 (green) and OX42 (red) staining in the spinal dorsal horn of the control side (**a**, **c**, **e**) and ipsilateral to transection (**b**, **d**, **f**) of the saphenous nerve in the L3 segment of the spinal cord. Note the disappearance of IB4 staining of C-fiber primary afferent terminals from somatotopically relating projection areas of the saphenous nerve (**b**, **f**). The accumulation of microglial cells is confined to the “IB4 gap” in laminae I–II and to the regions of the deeper laminae just ventrally to the “IB4 gap.” The scale bar in (**f**) indicates 100 μm and holds for all photomicrographs

Staining for IB4, a specific neurochemical marker of unmyelinated C-fiber primary sensory neurons, was used to demonstrate the distribution of C-fiber primary afferents in the spinal dorsal horn. In accord with previous reports (Silverman and Kruger 1990; Wang et al. 1994), the most intense labeling was detected in inner Rexed’s lamina II with some staining in lamina I (Fig. 1a). In agreement with previous findings (Shehab et al. 2004; Beggs and Salter 2007; Shehab 2014), transection of the sciatic nerve resulted in a practically complete abolition of IB4-staining in the somatotopically relating areas of the substantia gelatinosa (Fig. 1b). Transection of the sciatic nerve, a mixed nerve containing large populations of cutaneous and muscle afferents, resulted in robust microgliosis in the ipsilateral dorsal horn, characterized by a marked increase in both the density and intensity of OX42-immunoreactive microglial cells. In the superficial laminae I–II, microgliosis was confined to the somatotopically relating termination sites of the affected sciatic C-fiber afferents as revealed by the gap in the IB4-positive band indicating a strong topographical similarity between the projection territory of the (injured) peripheral nerve and the spatial extent of microgliosis (Fig. 1b, d, f). Microglia activation was also evident in the deeper laminae III–IV ventral to the IB4 gap (Fig. 1b–f). In identical (mirror) areas of the contralateral control side, no change in microglial density was observed in the central projection fields of the sciatic nerve (Fig. 1c, e). In the ventral horn, intense microglia reaction was observed around motoneurons ipsilateral to sciatic nerve transection (Fig. 1h). Similar microgliosis was observed in the somatotopically relating areas of the spinal dorsal horn after transection of the saphenous nerve, a cutaneous nerve (Fig. 2). Accordingly, peri-motoneuronal microgliosis was not observed in these rats (data not shown).

Quantitative data shown in Table 1 revealed a more than three-fold increase in microglial density, as expressed in percent area covered by OX42-positive profiles, in the superficial laminae I–II of the dorsal horn following sciatic nerve transection in comparison with the control side (control: 2.46 ± 0.51%, transected: 9.73 ± 1.28%). In the deeper laminae III–IV, the changes proved to be similar in intensity (control: 2.26 ± 0.98%; transected: 9.80 ± 1.72%). After transection of the saphenous nerve, microglia density increased more than two-fold compared with the control side in laminae I–II (control: 3.63 ± 1.36%; transected: 8.52 ± 2.75%), and about three-fold in the deeper laminae III–IV (control: 2.52 ± 0.87%; transected: 7.82 ± 2.66%). Sham operation did not significantly affect spinal microglial density (Table 1).

**Table 1:** Quantitative evaluation of microglial density in the spinal dorsal horn after peripheral nerve manipulations and the effect of prior capsaicin treatment thereon

	Laminae I–II		Laminae III–IV	
Treatment	Contra (%)	Ipsi (%)	Contra (%)	Ipsi (%)
Sciatic transection (*n* = 4)	2.46 ± 0.51	9.73 ± 1.28^*^	2.26 ± 0.98	9.80 ± 1.72^*^
Sciatic capsaicin (*n* = 4)	2.10 ± 0.44	2.80 ± 0.43	1.47 ± 0.58	2.16 ± 0.68
Sciatic capsaicin + transection (*n* = 4)	1.65 ± 0.45	9.08 ± 1.76^*^	1.01 ± 0.28	10.39 ± 2.05^*^
Saphenous transection (*n* = 4)	3.63 ± 1.36	8.52 ± 2.75^*^	2.52 ± 0.87	7.82 ± 2.66^*^
Saphenous capsaicin (*n* = 4)	3.77 ± 1.39	4.93 ± 1.35	1.76 ± 1.53	2.41 ± 1.53
Neonatal capsaicin + saphenous transection (*n* = 4)	2.85 ± 0.83	7.06 ± 1.83^*^	1.88 ± 0.75	7.09 ± 1.52^*^
Sham operations^#^ (*n* = 3)	1.57 ± 0.61	1.71 ± 0.60	1.03 ± 0.28	1.56 ± 0.57
Vehicle treatment^#^ (*n* = 4)	1.87 ± 0.38	2.28 ± 0.51	1.14 ± 0.31	1.81 ± 0.89

^*^Significantly different from the corresponding contralateral control value, *p* < 0.05

^#^Values obtained from sham-operated and vehicle-treated animals, respectively, did not differ significantly, and, therefore, they are shown as single treatment groups

### Effect of perineural application of capsaicin on the distribution and density of spinal microglial cells

**Fig. 3:**
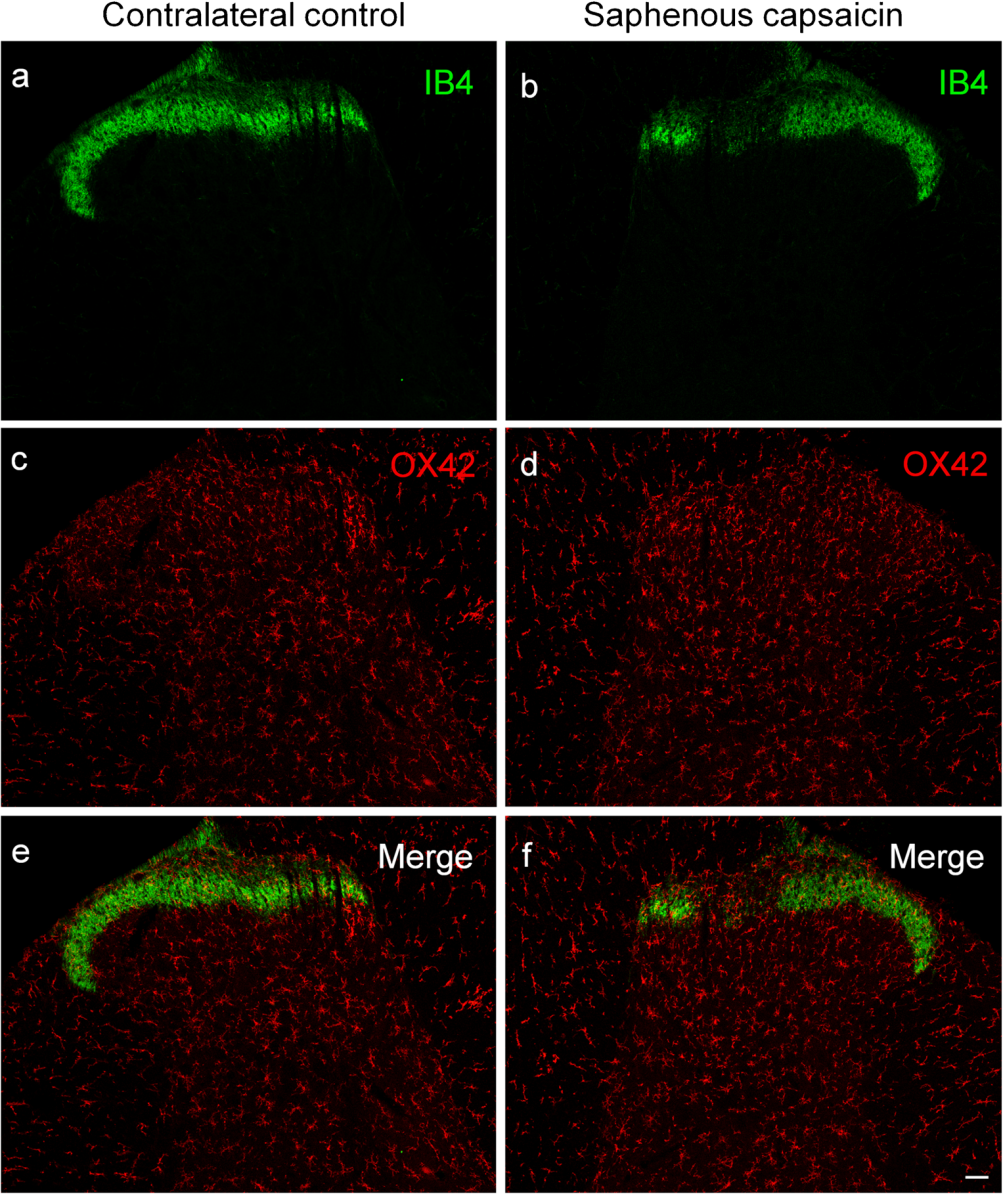
Fluorescent photomicrographs illustrating IB4 (green) and OX42 (red) staining in the spinal dorsal horn of the control side (**a**, **c**, **e**) and ipsilateral to perineural capsaicin treatment (**b**, **d**, **f**) of the saphenous nerve in the L3 segment of the spinal cord. Note the disappearance of IB4 staining of C-fiber primary afferent terminals from somatotopically relating projection areas of the sciatic nerve (**b**, **f**). There is no sign of accumulation of microglial cells neither in the superficial nor in the deeper laminae of the ipsilateral spinal dorsal horn. The scale bar in (**f**) indicates 100 μm and holds for all photomicrographs

**Fig. 4:**
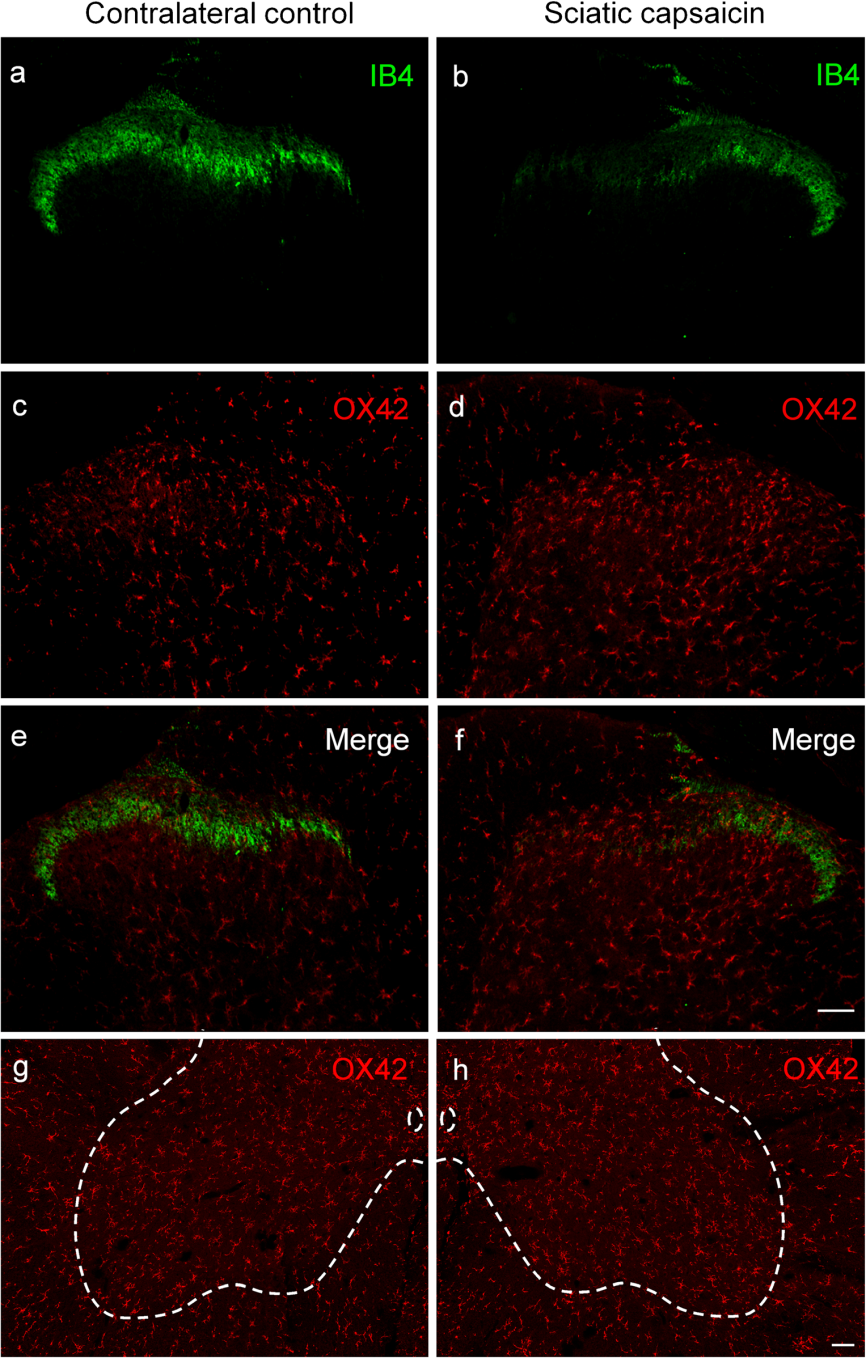
Fluorescent photomicrographs illustrating IB4 (green) and OX42 (red) staining in the L4 segment of the spinal cord dorsal or ventral horn ipsilateral (**b**, **d**, **f**, **h**) and contralateral (**a**, **c**, **e**, **g**) to perineural capsaicin treatment of the sciatic nerve. Note the disappearance of IB4 staining of C-fiber primary afferent terminals from somatotopically relating projection areas of the sciatic nerve (**b**, **f**). There is no sign of accumulation of microglial cells neither in the superficial nor in the deeper laminae of the ipsilateral spinal dorsal horn (**d**, **f**). There is no sign of perineural microglia reaction in the ventral horn ipsilateral to the treatment (**h**). The scale bars in (**f**) and (**h**) indicate 100 μm and hold for the photomicrographs (**a**–**f**) and (**g**–**h**), respectively

In agreement with earlier studies showing a highly selective effect of capsaicin on unmyelinated C-fiber nociceptive primary afferent neurons (Jancsó et al. 1977; Jancsó et al. 1980; Jancsó and Lawson 1990), and similar to nerve transection, IB4-binding was abolished in the somatotopically relating territories of the saphenous and sciatic nerves, respectively, after perineural treatment with capsaicin (Fig. 3b, Fig. 4b). In contrast to nerve transection, however, little if any increase in microglial density was observed in the spinal dorsal horn after perineural treatment with capsaicin (Fig. 3d, f, Fig. 4d, f). In addition, no change in OX-42 staining was observed in the ventral horn after perineural treatment of the sciatic nerve with capsaicin (Fig. 4h). Sham operation (perineural application of the vehicle for capsaicin) did not significantly affect spinal microglial density (Table 1). The quantitative data supported these microscopic observations and are shown in Table 1.

### Effect of neonatal capsaicin treatment on spinal microgliosis induced by peripheral nerve transection

**Fig. 5:**
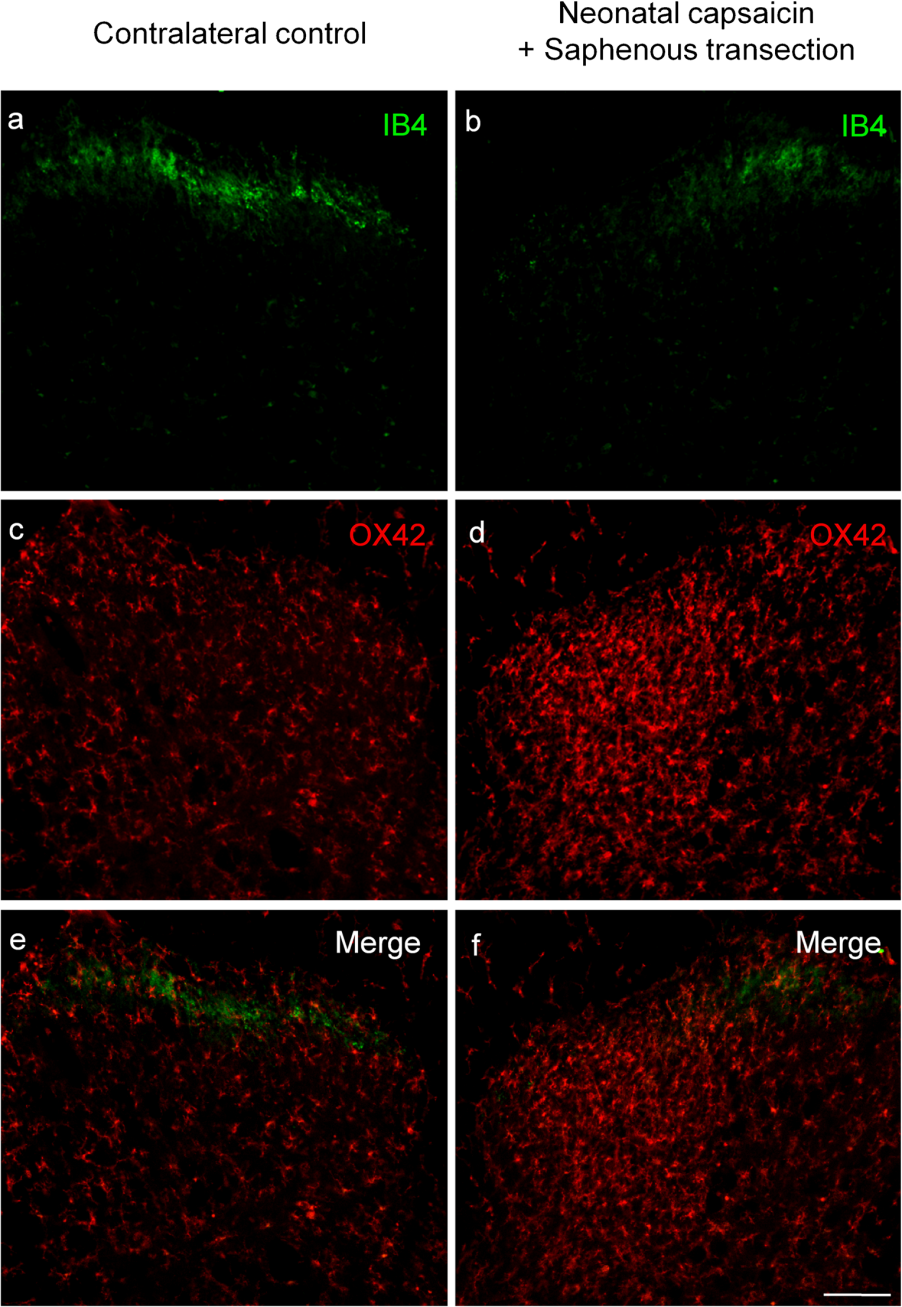
Fluorescent photomicrographs illustrating IB4 (green) and OX42 (red) staining in the spinal dorsal horn of the L3 segment of a rat treated neonatally with capsaicin. Note the strong reduction of IB4 staining on the control side (**a**, **e**) and the complete disappearance of IB4 staining of C-fiber primary afferent terminals in projection areas of the transected saphenous nerve (**b**, **f**). The marked increase in microglial density is confined to the area of the “IB4 gap” in laminae I–II and to regions of the deeper laminae just ventrally to the “IB4 gap.” The scale bar in (**f**) indicates 100 μm and holds for all photomicrographs

Neonatal treatment with capsaicin resulted in a massive decrease of IB4 binding in the substantia gelatinosa of the spinal dorsal horn in accord with the selective degeneration of C-fiber primary afferents after such treatment (Jancsó et al. 1977; Jancsó and Király 1980; Ritter and Dinh 1988). Transection of the saphenous nerve in neonatally capsaicin-treated rats abolished residual IB4 binding (Fig. 5b, f) parallel with the appearance of intense OX42 staining in the somatotopically relating areas of the superficial (laminae I–II) and deeper (laminae III–IV) layers of the spinal dorsal horn (Fig. 5d, f). The injury-induced microglial reaction was fully comparable in both extent and intensity with that obtained in naïve rats after peripheral nerve transection. The quantitative data support these observations and are shown in Table 1. Transection of the sciatic nerve yielded results similar to those obtained in naïve animals (data not shown).

### Effect of prior perineural application of capsaicin on spinal microgliosis induced by peripheral nerve transection

**Fig. 6:**
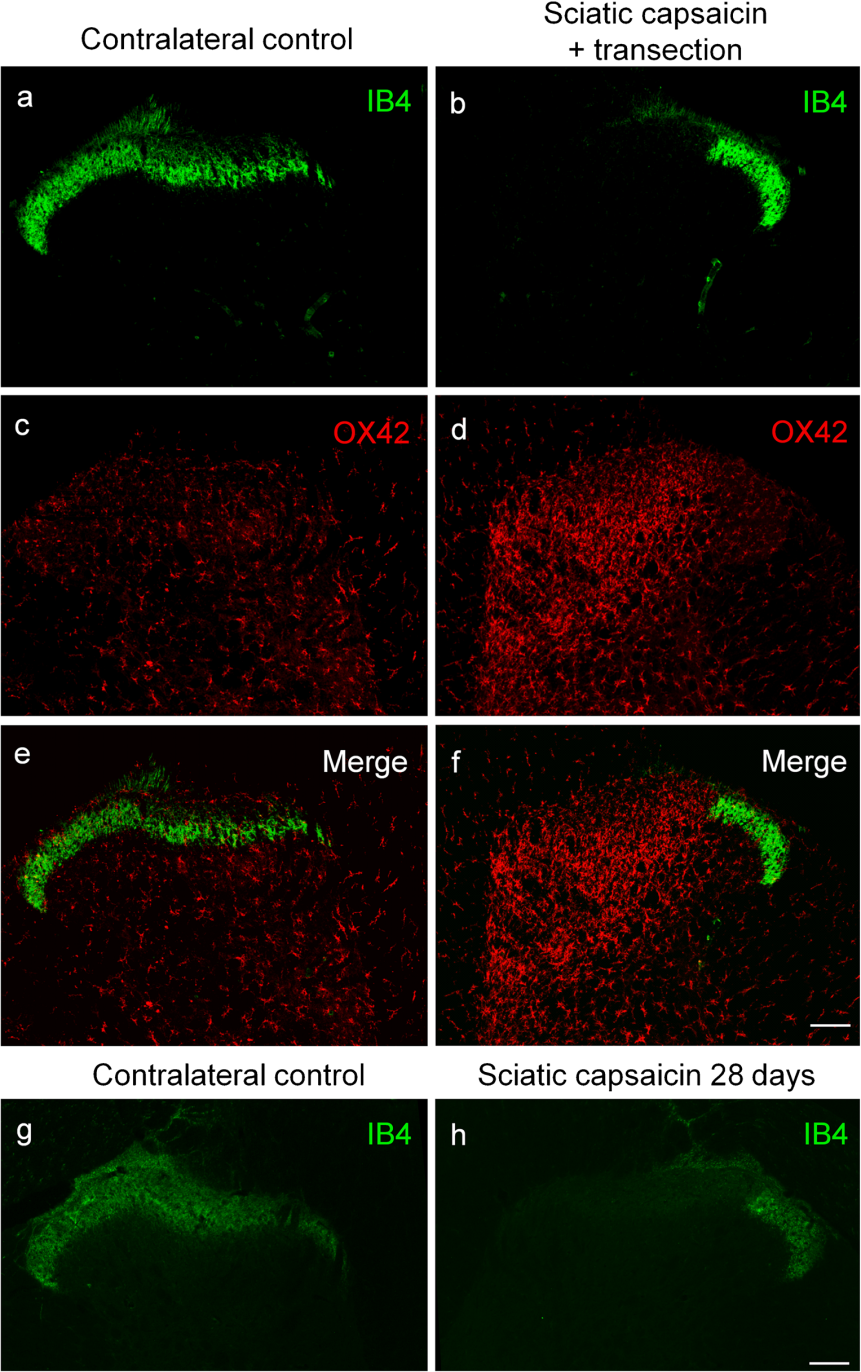
Fluorescent photomicrographs illustrating IB4 (green) and OX42 (red) staining in the L4 spinal dorsal horn of the control side (**a**, **c**, **e**) and ipsilateral to the transection (**b**, **d**, **f**) of the sciatic nerve, which has been chemically denervated by capsaicin 14 days previously. Note the disappearance of IB4 staining of C-fiber primary afferent terminals from somatotopically relating projection areas of the transected sciatic nerve (**b**, **f**) and the marked increase in microglial density (**d**, **f**). The spatial extent and intensity of microgliosis is fully comparable with that observed in naive animals. Twenty eight days after perineural capsaicin treatment, the spatial extent of the IB4 gap is comparable to that observed 14 days after perineural capsaicin (**h**). The scale bars in (**f**) and (**h**) indicate 100 μm and hold for the photomicrographs (**a**–**f**) and (**g**–**h**), respectively

In these experiments, perineural capsaicin treatment was performed to study peripheral nerve transection-induced spinal microgliosis following the complete functional elimination of C-fiber primary sensory neurons. Perineural application of capsaicin has been shown to abolish sensitivity to noxious heat and chemical irritants resulting in a practically complete defunctionalization of capsaicin-sensitive C-fiber primary sensory neurons (Jancsó et al. 1980; Fitzgerald and Woolf 1982; Gamse et al. 1982; Chung et al. 1985). Transection of a peripheral nerve 14 days after perineural capsaicin treatment resulted in robust spinal microgliosis confined to the somatotopically relating areas of the affected nerves (Fig. 6d, f) as assessed by the capsaicin treatment-induced appearance of gaps in the IB4-positive band of the substantia gelatinosa (Fig. 6b, f). This gap was a result of the capsaicin treatment since it can be observed in rats whose sciatic nerve was treated with capsaicin 28 days before sacrifice (Fig. 6h). The microglia reaction was comparable in both spatial extent and intensity to that seen after nerve transection in naïve rats. The quantitative data support these observations and are presented in Table 1.

## Discussion

Spinal microgliosis is a conspicuous manifestation of injuries inflicted upon peripheral nerves and may bear of crucial importance as regards the development of neuropathic pain (Gilmore and Skinner 1979; Sandkühler 2009; Calvo and Bennett 2012; Ji et al. 2013). Recent findings suggested the involvement of different chemokines released from peripherally injured spinal primary afferents in the mechanism of microglial activation (Maciejewski-Lenoir et al. 1999; Verge et al. 2004; Thacker et al. 2009; Sandkühler 2009; Kettenmann et al. 2011; Biber et al. 2011; Calvo and Bennett 2012; Ji et al. 2013; Clark and Malcangio 2014; Guan et al. 2015). However, knowledge on the types of primary afferent fibers that participate in the development of this phenomenon is limited. The present study addressed this question by utilizing selective chemical deletion techniques producing complete defunctionalization or near-total ablation of capsaicin-sensitive C-fiber primary afferent neurons (Jancsó et al. 1977; Gamse et al. 1982; Buck and Burks 1986; Holzer 1991). In agreement with previous findings, transection of the sciatic or saphenous nerve resulted in a dramatic increase in microglial cell density in the spinal dorsal horn. In the present study, we provided quantitative data on spinal microgliosis by measuring microglial density in the central projection territories of the injured nerves as assessed with IB4 lectin histochemistry. IB4 is a reliable and specific marker of unmyelinated nerve fibers (Silverman and Kruger 1990) which binds to a specific glycoprotein localized in small primary sensory neurons, including their central spinal terminals (Wang et al. 1994). In the spinal dorsal horn, IB4 binding is localized mostly to the substantia gelatinosa (Rexed’s lamina II) forming a continuous band along the entire medio-lateral aspect of the dorsal horn (Wang et al. 1994; Snider and McMahon 1998; Beggs and Salter 2007). Importantly, IB4 binding is function-dependent, since injuries to peripheral nerves result in the depletion of IB4 staining in the somatotopically relating areas of the substantia gelatinosa of the injured nerve (Shehab et al. 2004; Beggs and Salter 2007). This phenomenon was utilized in the present study to identify central projection territories of the injured nerves. Quantitative evaluation showed an about 3-fold increase of microglial density in both the most superficial (laminae I and II) and deeper (laminae III and IV) layers of the spinal dorsal horn after peripheral nerve transection. In accord with previous studies (Gilmore and Skinner 1979; Colburn et al. 1997; Zhang and De Koninck 2006), after transection of the sciatic nerve, a mixed nerve containing also motor fibers, an intense pericellular microglial reaction was noted around motoneurons, too. In sharp contrast, 14 days after perineural capsaicin treatment of the saphenous or the sciatic nerve, changes in microglial density were minimal or absent. Furthermore, 14 days after transection of a previously capsaicin-treated nerve, microgliosis in all affected regions of the spinal cord was comparable to that seen after nerve transection in naïve animals. Perineural treatment with capsaicin or resiniferatoxin has been shown to produce a complete defunctionalization of capsaicin-sensitive C-fiber primary afferents (Jancsó et al. 1980; Fitzgerald and Woolf 1982; Chung et al. 1985; Baranowski et al. 1986; Kissin et al. 2005; Kissin 2008) associated with a delayed partial loss of unmyelinated sensory fibers (Jancsó and Lawson 1990; Pini et al. 1990; Jancsó 1992). Noteworthy, within the time frame of the present study, spinal primary afferent terminations are preserved after perineural capsaicin treatment (Jancsó and Sántha 2004; Oszlács et al. 2015; Javed et al. 2020). Indeed, transganglionic transport of wheat germ agglutinin-horse radish peroxidase conjugate, a C-fiber afferent-specific retrograde tracer, and choleratoxin B subunit-horse radish peroxidase conjugate, another retrograde tracer taken up and transported by myelinated and injured C-fiber afferents, has been demonstrated to somatotopically relating projection territories of the capsaicin-treated sciatic nerve (Sántha and Jancsó 2003; Jancsó and Sántha 2004; Oszlács et al. 2015).

In contrast, neonatal treatment with capsaicin results in an almost complete elimination of C-fiber primary afferents from the spinal dorsal horn resulting in a selective C-fiber de-afferentation (Jancsó et al. 1977; Jancsó and Király 1980; Nagy et al. 1981; Ritter and Dinh 1988). Therefore, the findings of the present study showing little if any change in spinal microglial density after perineural capsaicin strongly suggest that C-fiber primary afferents do not play a significant role in the initiation or maintenance of the peripheral nerve injury-induced spinal microgliosis. Our observations in rats treated neonatally with capsaicin, demonstrating an unchanged microglial reaction in both extent and intensity after peripheral nerve transection, further support this conclusion. Taken together, these findings suggest that neither chemodenervation by perineural capsaicin nor prior elimination by neonatal capsaicin treatment of nociceptive C-fiber primary sensory neurons affect significantly the microglial reaction to peripheral nerve injury.

The present observations demonstrating that C-fiber primary sensory neurons do not significantly contribute to the peripheral nerve injury-induced central microgliosis is somewhat surprising for several reasons. First, C-fiber primary afferent neurons synthesize, transport, and release different neuropeptides from their central (and peripheral) nerve terminals upon peripheral stimulation or damage to their peripheral axons. These include substance P and calcitonin gene-related peptide, which have been shown to activate microglial cells (Carniglia et al. 2017). Perineural treatment with capsaicin or resiniferatoxin has been shown to produce neurochemical changes resembling in many respects nerve injury (Gibson et al. 1982; Gamse et al. 1982; Csillik 1987; Knyihár-Csillik et al. 1989; Jancsó and Lawson 1990; Jancsó 1992; Jancsó and Ambrus 1994; Hökfelt et al. 1994; Beggs and Salter 2007; Jancsó et al. 2012; Szigeti et al. 2012; Oszlács et al. 2015). Moreover, perineural application of capsaicin produces a blockade of axoplasmic transport (Jancsó et al. 1980; Gamse et al. 1982), similar to colchicine, which has been shown to evoke a microglial response in the spinal cord (Colburn et al. 1997). Second, this particular class of nociceptive sensory ganglion cells has been shown to express or upregulate, in response to injury, a variety of chemokines and other agents that produce microglia proliferation in the spinal cord. Neuregulin-1 is released from primary afferent terminals in the spinal dorsal horn and via activation of microglial erbB2 receptors stimulates proliferation and chemotaxis of microglia cells (Calvo et al. 2010). ATP released from primary afferents (Burnstock and Sawynok 2010) has also been implicated in injury-induced microglial chemotaxis via P2Y and P2X receptors (Honda et al. 2001). Many chemokines, such as CCL2, fractalkine (CX3CL1), and CCL21, mediate neuron-microglial interactions. Fractalkine has been implicated in injury-induced microglia activation, since it is expressed in spinal and dorsal root ganglion neurons and its receptor CX3CR1 is upregulated in microglia following nerve injury (Verge et al. 2004; Zhuang et al. 2007; Clark and Malcangio 2014). Peripheral nerve injury has been shown to increase the expression of chemokine CCL2 in small primary afferent neurons which is then transported to the spinal dorsal horn where it induces microglial activation associated with mechanical allodynia (Thacker et al. 2009). Recent findings, however, suggest that although CCL2 may attract macrophages into the spinal cord, only CCL21, expressed and transported into the spinal dorsal horn by peripherally injured primary afferent neurons, participates in neuron microglia signaling and triggers microglia activation via the P2X4 receptor (Biber et al. 2011; Biber and Boddeke 2014). Metalloprotease MMP-9 is also upregulated by nerve injury and transported into the spinal dorsal horn in calcitonin gene-related peptide-containing primary afferents, which are mostly nociceptive in function, to induce activation of spinal microglial cells (Kawasaki et al. 2008). Recent observations demonstrated that colony stimulating factor 1 expressed in both small and large dorsal root ganglion neurons after peripheral nerve injury is necessary and sufficient to elicit spinal microgliosis and mechanical hypersensitivity (Guan et al. 2015). The present findings may suggest that, in small dorsal root ganglion neurons, peripheral nerve injury-induced expression of different agents, including chemokines and colony stimulating factor 1, may be a cellular response to nerve damage but, interestingly, may not contribute significantly to spinal microgliosis. Indeed, peripheral nerve injury invariably elicited marked spinal microgliosis in both extent and intensity after complete defunctionalization or near total elimination of C-fiber primary afferents. Therefore, the findings presented in this study strongly indicate that C-fiber primary sensory neurons may bear of limited importance in initiating or maintenance of spinal microgliosis following peripheral nerve injury. The findings of the present study are in line and give further support for the notion that selective blockade by resiniferatoxin of activation of unmyelinated nociceptive afferent nerve fibers failed to affect the spinal microglial reaction (Suter et al. 2009). In both a previous (Suter et al. 2009) and the present study, TRPV1 agonists, resiniferatoxin and capsaicin, respectively, were used to block and/or eliminate C-fiber afferents. These highly potent TRPV1 agonists may block/eliminate an overwhelming majority of unmyelinated afferent fibers with only a very small capsaicin-insensitive population remaining. Neonatal capsaicin treatment has been shown to result in a loss of up to 95% of unmyelinated dorsal root axons (Nagy et al. 1981), whereas perineural treatment with capsaicin produces a complete functional (Jancsó et al. 1980; Fitzgerald and Woolf 1982; Gamse et al. 1982; Chung et al. 1985; Baranowski et al. 1986; Jancsó and Lawson 1987; Pini and Lynn 1991; Jancsó et al. 2008; Jancsó et al. 2012) and conduction block (Petsche et al. 1983; Such and Jancsó 1986; Jancsó et al. 1987; Pini and Lynn 1991; Kissin et al. 2005; Suter et al. 2009) of C-fibers in peripheral nerves. Hence, utilization of potent TRPV1 agonists, such as capsaicin and resiniferatoxin, producing a selective C-fiber sensory neuron blocking effect, proved to be a reliable approach to study the contribution of this particular type of nociceptive primary afferent neurons to peripheral nerve injury-induced pathologies, including spinal microgliosis.

In conclusion, the present experiments demonstrated that functional blocking or elimination of C-fiber primary afferent neurons failed to significantly interfere with the development and maintenance of peripheral nerve injury-induced increase in spinal microglial density. These findings strongly suggest that development of the microglial response and, in turn, neuropathic pain behavior may be largely dependent on the activation of and changes in the expression profile of myelinated primary afferents. This is supported by electrophysiological findings demonstrating that ectopic discharges originating from injured afferent axons play an important role in the development of neuropathic pain (Wall and Gutnick 1974) and ectopic discharges recorded from injured peripheral nerve originated from Aβ and Aδ axons with a very minor contribution from C-fiber afferents (Han et al. 2000). Results of more recent studies using selective optogenetic stimulation of different types of Aβ primary afferents and demonstrating that it is both necessary and sufficient for producing pain from light touch after nerve injury in transgenic mice (Dhandapani et al. 2018) and rats (Tashima et al. 2018) are in line with these findings. Another study suggested that optogenetic stimulation of a subpopulation of Aβ primary afferents may not be sufficient to induce tactile allodynia in mice after nerve injury, which, however, might be explained by lack of stimulation of critical populations of Aβ afferents (Chamessian et al. 2019).

Local application of capsaicin or resiniferatoxin onto sensory ganglia or peripheral nerves has been proved to produce significant antinociceptive effect in different animal models of neuropathic and cancer pain (Jancsó and Lynn 1987; Lynn 1990; Karai et al. 2004; Kissin 2008; Brown et al. 2015), possibly through the regulation of TRPV1 receptor expression (Szigeti et al. 2012). The present findings suggest that this new type of “nociceptor analgesia” (Karai et al. 2004; Kissin 2008; Jancsó et al. 2008; Jancsó et al. 2012) is not associated with phenomena involving microglial activation.

The present study elucidating the role of different classes of primary afferent neurons in the development of reactive spinal microgliosis by introducing specific and selective pharmacological techniques may help to find further clues for the understanding of the mechanisms of peripheral nerve injury-induced spinal changes, including microgliosis. These observations also suggest that the integrity of myelinated primary afferents may play a role in the maintenance of the neuronal, glial, and immune microenvironment, and possibly structural organization of the spinal cord.
